# ZIKV Demonstrates Minimal Pathologic Effects and Mosquito Infectivity in Viremic Cynomolgus Macaques

**DOI:** 10.3390/v10110661

**Published:** 2018-11-21

**Authors:** Sasha R. Azar, Shannan L. Rossi, Sherry H. Haller, Ruimei Yun, Jing H. Huang, Jessica A. Plante, Jiehua Zhou, Juan P. Olano, Christopher M. Roundy, Kathryn A. Hanley, Scott C. Weaver, Nikos Vasilakis

**Affiliations:** 1 Department of Pathology, University of Texas Medical Branch, Galveston, TX 77555, USA; srazar@utmb.edu (S.R.A.); slrossi@utmb.edu (S.L.R.); jinghong2018@yahoo.com (J.H.H.); jiehzhou@utmb.edu (J.Z.); jolano@utmb.edu (J.P.O.); 2 Department of Microbiology and Immunology, University of Texas Medical Branch, Galveston, TX 77555, USA; shhaller@utmb.edu (S.H.H.); ruyun@utmb.edu (R.Y.); japlante@utmb.edu (J.A.P.); cmroundy@utmb.edu (C.M.R.); 3 Institute for Translational Sciences, University of Texas Medical Branch, Galveston, TX 77555, USA; 4 Institute for Human Infections and Immunity, University of Texas Medical Branch, Galveston, TX 77555, USA; 5 World Reference Center for Emerging Viruses and Arboviruses, University of Texas Medical Branch, Galveston, TX 77555, USA; 6 Department of Biology, New Mexico State University, Las Cruces, NM 88003, USA; khanley@nmsu.edu

**Keywords:** *Macaca fascicularis*, Zika virus, *Aedes aegypti*, pathogenesis, vector competence, Flaviviridae

## Abstract

To evaluate the effects of ZIKV infection on non-human primates (NHPs), as well as to investigate whether these NHPs develop sufficient viremia to infect the major urban vector mosquito, *Aedes aegypti*, four cynomolgus macaques (*Macaca fascicularis*) were subcutaneously infected with 5.0 log_10_ focus-forming units (FFU) of DNA clone-derived ZIKV strain FSS13025 (Asian lineage, Cambodia, 2010). Following infection, the animals were sampled (blood, urine, tears, and saliva), underwent daily health monitoring, and were exposed to *Ae. aegypti* at specified time points. All four animals developed viremia, which peaked 3–4 days post-infection at a maximum value of 6.9 log_10_ genome copies/mL. No virus was detected in urine, tears, or saliva. Infection by ZIKV caused minimal overt disease: serum biochemistry and CBC values largely fell within the normal ranges, and cytokine elevations were minimal. Strikingly, the minimally colonized population of *Ae. aegypti* exposed to viremic animals demonstrated a maximum infection rate of 26% during peak viremia, with two of the four macaques failing to infect a single mosquito at any time point. These data indicate that cynomolgus macaques may be an effective model for ZIKV infection of humans and highlights the relative refractoriness of *Ae. aegypti* for ZIKV infection at the levels of viremia observed.

## 1. Introduction

Zika virus (ZIKV), a mosquito-borne virus of the family *Flaviviridae*, was originally discovered in 1947 from the blood of a febrile sentinel rhesus macaque from Uganda’s Ziika forest [1]. Based on early animal models [2] and laboratory infection of a human volunteer [3], ZIKV appeared to cause only mild disease. The scant number of human infections reported in the medical literature suggested that, in Africa, ZIKV circulated only in a sylvatic, enzootic cycle, in which forest-dwelling mosquitoes such as *Aedes (Stegomyia) africanus* Theobald and *Ae. (Diceromyia) furcifer* Edwards transmitted the virus among nonhuman primates such as patas (*Erytrocebus patas*) and African green monkeys (*Chlorocebus aethiops*) [4,5,6,7,8], with occasional human infections resulting from spillover. Additionally, ZIKV may have been circulating in Southeast Asia as early as the 1950s [9,10,11], although it was not associated with reports of clinical disease. Due to co-circulation of other flaviviruses in Southeast Asia (dengue [DENV], West Nile [WNV], Japanese encephalitis [JEV], etc.), it is hypothesized that cross-reactive immunity in this population forestalled extensive ZIKV outbreaks [7,9,12].

The first contemporary large-scale outbreaks of ZIKV took place in 2007, taking place in Gabon as well as on the Island of Yap in the Federated States of Micronesia, with the latter outbreak infecting a substantial proportion of the island’s population [13,14]. Following the 2007 outbreaks, ZIKV spread across the islands of the South Pacific, akin to the spread of chikungunya virus (CHIKV) [15]. During this time, ZIKV caused outbreaks in French Polynesia, New Caledonia, Easter Island and the Cook Islands [15,16], before reaching Brazil by early 2013 [17,18,19]. In the Americas, detection of human illness and autochthonous mosquito transmission took place in early 2015 [15,20,21], preceding the explosive outbreak that is estimated to have infected between 400,000 and 1.3 million individuals in Brazil and spread to over 50 countries and or territories by March of 2016 [22].

These recent outbreaks validate early findings that human infection with ZIKV is largely asymptomatic [23,24,25,26]. Symptomatic ZIKV infections produce a nonspecific febrile illness (Zika fever [ZIKF]), characterized by fever, headache, conjunctivitis, myalgia, arthralgia and a maculopapular rash, which is difficult to distinguish from the cocirculating arboviruses DENV and CHIKV [23,27]. Despite the seemingly mild clinical presentation of ZIKV infection, the magnitude of the 2015–2016 outbreaks allowed for the discovery of ZIKV’s potential to cause serious sequelae: Congenital Zika Syndrome (CZS) in infants whose mothers were infected during pregnancy, as well as ophthalmic and auditory complications in infants [28,29,30,31], Guillain–Barré syndrome (GBS) in many age groups [32,33,34], and persistent shedding of viable virus and RNA in semen of infected men months after the primary infection [35]. Of these, the shedding of virus in semen and other bodily fluids have been shown to mediate sexual transmission [36,37], further complicating control of ZIKV’s spread.

The severity of ZIKV-mediated sequelae and the scale of the outbreaks in the Americas spurred efforts to develop vaccines and therapeutics [38,39,40]. Although several vaccine candidates have advanced to clinical testing [41,42,43], none has been licensed. Therefore, the best methods to prevent human infections remain mosquito control and public education. Several mosquitoes in the genus *Aedes* have been implicated as ZIKV vectors among humans through field surveillance, epidemiological analyses, as well as experimental infections. As a result, *Ae*. (*Stegomyia*) *hensilli* Farner, *Ae.* (*Stegomyia*) *albopictus* Skuse and *Ae.* (Stegomyia) aegypti Linnaeus have been implicated in urban outbreaks on the Island of Yap and in Gabon, Chiapas State Mexico, and Brazil, respectively [7,14,44,45]. Experimental vector competence analyses have further substantiated the ability of *Ae. aegypti* and *Ae. albopictus* to transmit ZIKV following oral exposure, via both artificial bloodmeals and viremic rodents [46,47,48], as well as *Ae.* (*Ochlerotatus*) *vexans* Meigen following oral infection [49].

Experimental analyses of vector competence for ZIKV have utilized artificial bloodmeals or viremic mice, generally at higher titers (4–7 log_10_ focus forming units/mL) [46,47,48,49] than those reported in symptomatic humans (1–5 log_10_ genome copies/mL) [50,51,52,53]. In the absence of experimental infections using viremic humans (e.g., well-documented for DENV [54]), vector competence is best examined by exposure of mosquitoes to titers of ZIKV corresponding to human viremia. Although small animal models such as laboratory mice, can and have been used to initially assess pathogenesis of ZIKV and related viruses, as well as the efficacy of vaccines and therapeutics due to their efficiency and cost-effectiveness [55,56,57], a truer representation of human disease is often only observed in non-human primates (NHPs) [58,59,60,61]. Both cynomolgus (*Macaca fascicularis*) and rhesus (*Macaca mulatta*) macaques have been developed as ZIKV models to examine pathogenesis [62], virus strain/lineage comparisons [63,64], infection and immune dynamics [65,66,67], long-term viral persistence [68,69], viral replication in reproductive tissue/modes of sexual transmission [70,71], and in utero infection/CZS [72,73,74]. Both species generate lower peak viremia titers than those typically used in mosquito oral exposure experiments [62,63,64,65,66,67,68,69,70,71,72,73,74], although these titers are comparable to those reported in human disease [50,51,52,53]. To date, there is only a single report of ZIKV infection of *Ae. aegypti* through feeding on viremic macaques, involving a single experimentally infected mosquito [75]. Data on *Ae. aegypti* susceptibility to ZIKV following natural bloodmeals that approximate human levels of viremia are critical to the understanding of the virus’ ability to maintain itself in human-mosquito-human urban cycles.

In the present study, four adult cynomolgus macaques were subcutaneously inoculated with 5 log_10_ focus-forming units (FFU) of ZIKV (infectious clone-derived strain FSS13025, Asian Lineage, Cambodia, 2010) and underwent a 28-day monitoring period during which weight, temperature, and samples of blood and other body fluids (tears, urine, saliva) were collected. Cohorts of low generation (F4) *Ae. aegypti* were allowed to feed on anesthetized animals periodically to assess susceptibility. At the end of the study period, each animal was euthanized, and tissues were collected for examination of histopathologic changes and ZIKV persistence in sites such as the reproductive tract, central nervous system, and lymph nodes.

## 2. Materials and Methods

### 2.1. Animals, Study Design and Ethics Statement

**Figure 1:**
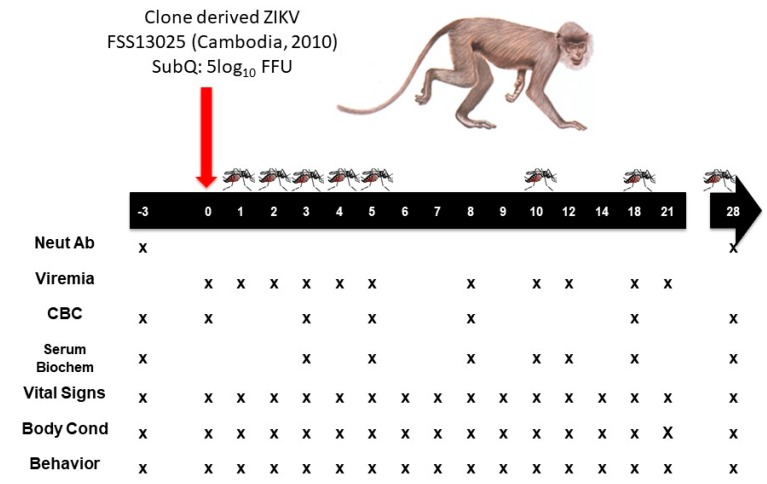
**Experimental design and timeline.** Monkeys were infected with 5 log_10_ FFU of cDNA clone-derived ZIKV strain FSS13025 on day 0. Samples (blood, urine, tears and saliva) were taken 3 days prior to infection and on 1, 2, 3, 4, 5,8, 10, 12, 18, 21, and 28 days post infection (dpi). All animals underwent terminal sampling and necropsy 28 dpi to detect any persistent infection or tissue damage due to infection.

Healthy adolescent (2–5-year-old) cynomolgus macaques (*Macaca fascicularis*) of Chinese origin were used: two males (0844 and 3368) and two females (3422 and 3428). All animals were seronegative for herpes B virus, simian immunodeficiency virus, simian retrovirus, and simian T-lymphotropic virus. Additional hemagglutinin inhibition testing performed on site upon arrival revealed no previous flavivirus [ZIKV, WNV, yellow fever (YFV), St. Louis encephalitis (SLEV), and all four DENV serotypes], or alphavirus [CHIKV, Venezuelan (VEEV), eastern (EEEV), or western equine encephalitis (WEEV), Sindbis (SINV), or Semliki forest virus (SFV)] exposure. Prior to ZIKV infection (≥6 months), these animals were exposed to CHIKV vaccines and challenged with wild-type CHIKV. All experiments were performed in full compliance with the guidelines established by the Animal Welfare Act for the housing and care of laboratory animals and conducted as laid out in University of Texas Medical Branch Institutional Animal Care and Use Committee (UTMB-IACUC) approved protocol (protocol #1702017, approved 13 March 2017). Animals were individually housed in steel caging (Allentown Inc., Allentown, NJ, USA) with free access to water. Commercial chow was provided twice daily, and supplementation was provided daily or at the discretion of the veterinary staff. Health checks were performed twice daily from the date of infection until the end of the study. Procedures and manipulations were conducted with care to minimize any potential distress, pain or discomfort by trained veterinary and/or research staff. On specified days (Figure 1) following an overnight fast, animals were anesthetized by intramuscular injection with Ketamine (5–20 mg/kg) then weighed, and temperatures measured via a rectal probe. Blood was collected by venipuncture into K_2_EDTA or serum separator vacutainer tubes (SSTs; BD, Franklin Lakes, NJ, USA). Urine was collected in caging pans, tears were collected by insertion of sterilized strips of Whatman filter paper (Sigma-Aldrich, St. Louis, MO, USA) into the lacrimal duct, and saliva was collected by oral swabbing with sterilized cotton swabs. These samples were immediately added to 500 μL of Dulbecco’s modified Eagle’s Medium (DMEM, Invitrogen, Carlsbad, San Diego, CA, USA) supplemented with 2% fetal bovine serum (FBS, Atlanta Biologicals, Norwalk, GA, USA) and Penicillin/Streptomycin (P/S; 100 Units/mL and 100 μg/mL respectively, Invitrogen). A 0.5 L cardboard carton with a mesh top containing sucrose-starved female *Ae. aegypti* was placed on the anesthetized NHP’s ear and mosquitos were allowed to feed for ≈10–15 min. Animals were euthanized at the end of the study by pentobarbital overdose followed by necropsy; brain, liver, kidney, spleen, spinal cord, eyes, nerve, draining lymph node and testes samples were collected and immediately immersed in formalin for fixation and histological analysis.

### 2.2. Cell Lines and Viruses

Vero cells (CCL-81) were purchased from the American Type Culture Collection (Bethesda, MD, USA) and maintained in DMEM supplemented with 5% FBS and penicillin/streptomycin (P/S; 100 Units/mL and 100 μg/mL respectively) at 37 °C with 5% CO_2_. An infectious cDNA clone of ZIKV-FSS13025 (GenBank number KU955593.1), was utilized to generate virus stocks as described previously [76].

### 2.3. Infection of Macaques

Dosages in the experiment were based on previous reports of ZIKV infection of cynomolgus macaques as well as *Ae. aegypti* salivary titers [62,66,75]. ZIKV FSS12035 harvested directly from electroporated Vero cells was diluted to 6.0 log_10_ FFU in sterile Dulbecco’s Phosphate Buffered Saline (DPBS, Invitrogen, Carlsbad, San Diego, CA, USA). Macaques were anesthetized as described above and were inoculated with 5.0 log_10_ FFU of ZIKV subcutaneously in a volume of 100 μL.

### 2.4. Titrations

Virus samples were assayed by 10-fold serial dilution in DMEM on Vero cell monolayers. After 1 h at 37 °C, wells were overlaid with 0.8% methylcellulose in DMEM. Following 3 days incubation at 37 °C, the overlay was removed and monolayers were rinsed twice with sterile DPBS, and fixed for 1 h at room temperature in ice-cold methanol:acetone (1:1). Detection of virus was conducted via focus-forming assay, as detailed below.

### 2.5. Focus-Forming Assay

Focus-forming assays (FFAs) were performed as previously described [48,77,78], with modifications. Viral dilutions or clarified mosquito homogenates were inoculated onto Vero cell monolayers on 12- or 96-well plates, respectively. Following a three-day infection, plates were washed and fixed as described above and stained using a mouse hyperimmune polyclonal anti-ZIKV primary antibody (World Reference Center for Emerging Viruses and Arboviruses, UTMB), and HRP-labeled goat anti-mouse secondary antibody (KPL, Gaithersburg, MD, USA). Detection was performed using an aminoethylcarbazole (AEC) solution (Enzo Diagnostics, Farmingdale, NY) prepared according to manufacturer’s protocol. For samples in 96-well plates, detection of any intracytoplasmic staining by light microscopy was defined as positive.

### 2.6. Plaque-Reduction Neutralization Titers

Neutralization/seroconversion was determined utilizing a standard 50% plaque reduction neutralization test (PRNT_50_) as previously described [79]. The neutralizing titer was represented as the reciprocal of the highest dilution of serum that inhibited 50% of foci (PRNT_50_). Samples scored below the limit of detection for a single plate (<1:20) were considered negative. Vero-passaged (Vero-1, C6/36-2, Vero-4) ZIKV FSS13025 isolate (WRCEVA, UTMB, Galveston, TX, USA) was utilized as the reference strain.

### 2.7. qRT-PCR Analysis

Viral RNA was isolated from whole blood, tears, saliva or urine using a viral RNA mini prep kit (Qiagen). ZIKV genomic RNA was assayed using virus-specific primers (Zika4481 CTG TGG CAT GAA CCC AAT AG and Zika4552c ATC CCA TAG AGC ACC ACT CC) and probe (Zika 4507cFAM CCA CGC TCC AGC TGC AAA GG) for qRT-PCR (BioRad iTaq Universal Probes One-Step kit [80]. Each sample was tested in duplicate, and average Ct values were calculated and converted to genome copies/mL based on a in-house standard curve generated from serially diluted RNA from an in vitro transcribed ZIKV FSS13025.

### 2.8. Preparation and Administration of Artificial Infectious Bloodmeals

For artificial bloodmeal mosquito experiments, clone-derived ZIKV strain FSS13025 stocks were serially diluted. Five bloodmeals with titers of approximately 4.3 log_10_, 5.3 log_10_, 6.3 log_10_, 7.3 log_10_, and 8.3 log_10_ genome copies/mL were prepared as previously described [47] and loaded into Hemotek feeding systems (Hemotek Ltd., Blackburn, UK) overlaid with laboratory paraffin film (Bemis, Oshkosh, WI, USA). Cohorts of sucrose-starved *Ae. aegypti* mosquitoes were allowed to feed for 1 h. Fully engorged mosquitos were incubated as described below.

### 2.9. Mosquitoes

Adult female *Ae. aegypti* from a Galveston colony (F3 for viremic macaques, F4 for artificial bloodmeals) were housed in a 27 ± 1 °C incubator at a 16:8 light:dark photoperiod with 80 ±10% relative humidity, fed 10% sucrose ad libitum, and maintained, sampled, and processed as described previously [47,48].

### 2.10. Serum Biochemistry and Complete Blood Counts

To determine hematological parameters, whole blood in K_2_EDTA tubes was analyzed using the Hemavet 950 system (Drew Scientific Inc., Miami Lakes, FL, USA) according to manufacturer instructions. For serum biochemical parameters, serum was harvested from SST tubes and clarified by centrifugation (5 min, 6000× *g*). Serum was immediately aliquoted and stored at −80 °C for PRNT and Bioplex assays. At least 200 μL from each NHP was not frozen and inoculated immediately into a Comprehensive Diagnostic Profile rotor (Abaxis, Union City, CA, USA) and subjected to biochemical analysis via the VetScan VS2 (Abaxis, Union City, CA, USA) according to manufacturer instructions.

### 2.11. Cytokine/Chemokine Assays

Cytokine and chemokine expression were quantified in serum taken 1, 3, 5, 12 and 21 days post-infection (dpi) as well as from 3 days prior to infection using the Milliplex NHP Cytokine/Chemokine Magnetic 23-Plex Panel (EMD Millipore, Billerica, MA, USA) according to manufacturer’s protocol in a Bio-Plex 200 system (BioRad, Hercule, CA, USA). Each sample was run in duplicate, and results for each animal are presented as fold change over the value observed in serum three days prior to infection using GraphPad Prism 6.0 software (GraphPad Software Inc., La Jolla, CA, USA).

### 2.12. Tissue Processing, Staining, and Analysis

Formalin-fixed paraffin-embedded tissues (spleen, liver, kidneys, eyes, brains, testes, nerves, and lymph nodes) were sectioned and stained by hematoxylin and eosin (H&E). De-identified glass slides were examined by a Board-Certified Anatomic pathologist alongside historical uninfected control samples (a generous gift from Sarah Lockwood, California National Primate Research Center).

### 2.13. Statistical Analysis

All statistics were conducted in JMP (SAS, Cary, NC, USA). Fisher’s exact tests were used to compare the proportion of mosquitoes with infection or disseminated infection in all two by two comparisons.

## 3. Results

### 3.1. Viral Loads in Bodily Fluids and Seroconversion

**Figure 2:**
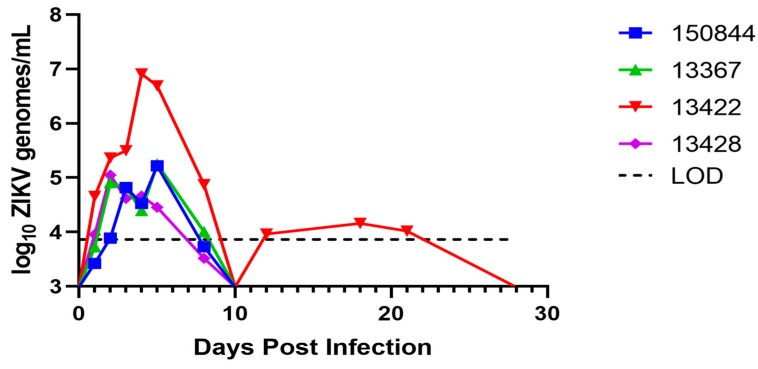
**Whole blood viral load.** Results of iTaq One-Step qRT-PCR to determine whole blood viral load in each animal on the specified day through conversion to genome copies/mL by standard curve. Macaque 150844 seen in blue, 13367 in green, 13422 in red and 13428 in purple. Limit of detection for the assay demonstrated at ≈3.9 genome copies/mL based on a ≈100 genome/mL whole blood detection limit and 140 µL extraction from whole blood.

To detect ZIKV, viral RNA was extracted from whole blood, urine, tears, and saliva on 1, 2, 3, 4, 5, 8, 10, 12, 18 and 28 dpi. Viral RNA was detected in the blood of all four macaques, although in three of the four animals (150844, 13367, and 13428), titers did not exceed 5.4 log_10_ genome copies/mL. The maximum viremia was observed in subject 13422, peaking at 6.9 log_10_ genome copies/mL 4 dpi. In three of the four animals (13422, 150844, and 13367), viremia peaked 4 or 5 dpi, while in monkey 13428, viremia peaked on 2 dpi. Viremia in all four macaques fell below the limit of detection (≈3.9 genome copies/mL) by 10 dpi. Viremia in macaque 13422 rebounded to a low level (≈4 log_10_ genome copies/mL) on 12, 18, and 21 dpi. By 28 dpi, viremia had fallen below the limit of detection in all animals (Figure 2). Despite multiple reports in the literature, ZIKV was not detected in urine, tears, or saliva, potentially due to dilution in carrier media. PRNT_50_ assays performed on pre-exposure revealed all NHPs maintained a neutralizing antibody response against CHIKV (PRNT_50_ ≥ 640) but no existing neutralizing response against ZIKV (PRNT_50_ < 20), while terminal serum demonstrated that all animals underwent seroconversion (ZIKV PRNT_50_ titers of 1:20 in macaques 13428 and 13367, 1:40 in macaque 13422 and 1:80 in macaque 150844).

### 3.2. Clinical Findings

**Figure 3:**
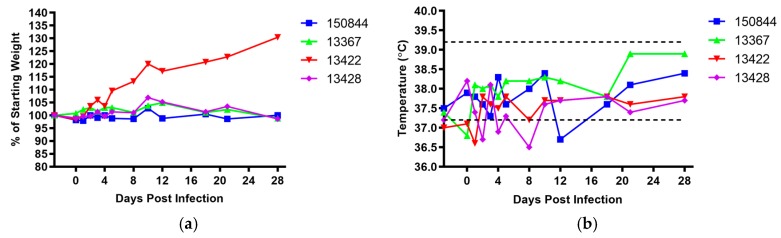
**Weight change and rectal temperatures.** Following anesthesia on specified days (−3, 0, 1, 2, 3, 4, 5, 8, 10, 12, 18, 21, and 28 dpi), animals were (**a**) weighed and (**b**) rectal temperatures were taken. Weights represented by % of individual animal’s weight on the day that study commenced (3 days prior to infection). Macaque 150844 seen in blue, 13367 in green, 13422 in red and 13428 in purple. Dotted lines in (**b**) represent upper and lower bounds of the range for normal cynomolgus macaque rectal temperatures [81].

Subsequent to infection, macaques were checked twice daily for any signs of morbidity such as loss of appetite/weight loss, dehydration, diarrhea, rash, and fever. None demonstrated overt weight loss over the course of the study, with subject 13422 actually increasing in weight, likely due to dietary enrichment and a lower than average starting weight (Figure 3a). No fever was observed, although all four macaques demonstrated mild hypothermia between 1 and 12 dpi (Figure 3b). No other clinical signs of disease were noted.

**Figure 4:**
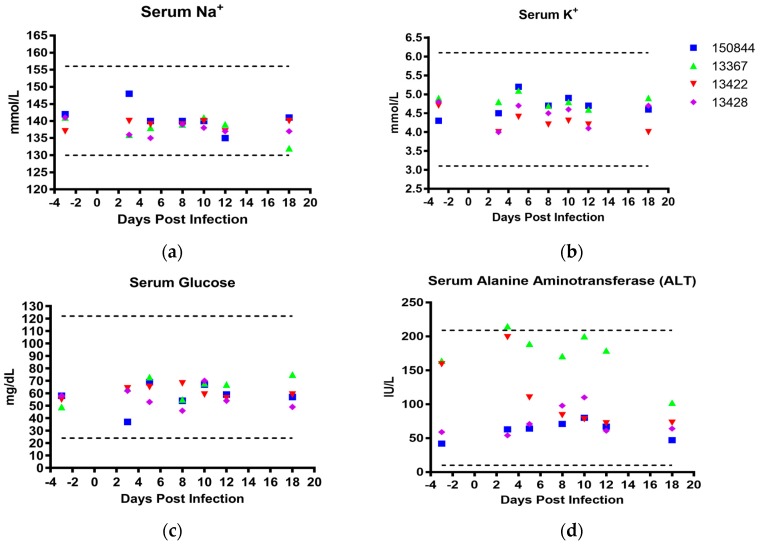
**Serum Biochemistries.** Sera was analyzed utilizing the VetScan VS2 system. (**a**) serum Na^+^ in mmol/L, (**b**) serum K^+^ in mmol/L, (**c**) serum glucose in mg/dL, and (**d**) serum alanine aminotransferase in IU/L. Macaque 150844 seen in blue, 13367 in green, 13422 in red and 13428 in purple. Dotted lines represent upper and lower reference ranges for analytes.

Serum biochemistries and complete blood counts (CBCs) were performed at specified days following infection. Reference values from the laboratory animal reference manual [81] were used to evaluate the results. No abnormalities in electrolyte (Na^+^, K^+^, Ca^2+^, and PO_4_^2−^, Figure 4a,b and Figure S1f,g) or glucose (Figure 4c) levels were observed at any time point except for macaque 150844, which demonstrated mildly elevated levels of PO_4_^2−^ on 5 and 12 dpi (Figure S1g). Additionally, a spike in serum alanine aminotransferase levels (ALT) in macaques 13367 and 13422 was observed 3 dpi, and decreased in subject 13422 two days later while ALT levels in macaque 13367 remained elevated until 18 dpi. (Figure 4d). Macaque 13422 also demonstrated a sharp increase in serum albumin 5 dpi. (Figure S1a), while 13367 and 13428 exhibited a spike in total bilirubin on 3 and 5 dpi and 3, 10, and 18 dpi respectively (Figure S1d). No other deviations outside of reference ranges or substantial alterations from pre-infection values were observed in other serum factors such as blood urea nitrogen, total protein or globulin (Figure S1e,i,h,j).

**Figure 5:**
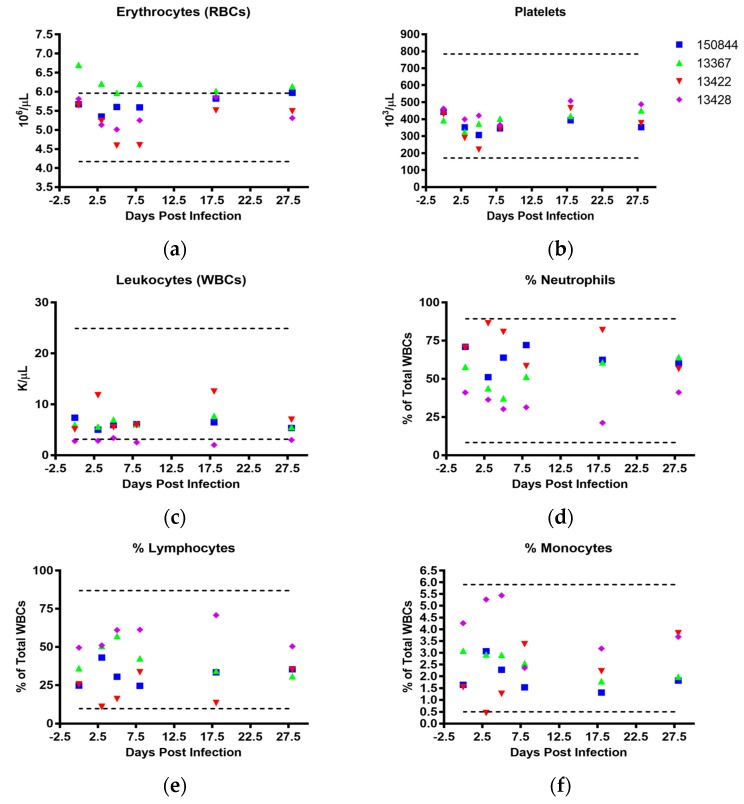
**Hematological Values.** Whole blood collected in K_2_EDTA tubes was utilized to perform complete blood counts using the HemaVet 950 system. (**a**) erythrocytes in 10^6^/µL, (**b**) platelets in 10^3^/µL, (**c**) leukocytes in K/µL, (**d**) neutrophils as % of total leukocytes, (**e**) lymphocytes as percentage of total leukocytes, and (**f**) monocytes as % of total leukocytes. Macaque 150844 seen in blue, 13367 in green, 13422 in red and 13428 in purple. Dotted lines represent upper and lower reference ranges for analytes.

With respect to CBCs, subject 13367 also demonstrated elevated red blood cell (RBC) counts prior to infection, which stayed slightly above the reference range for the course of the study (Figure 5a). Although this animal exhibited levels of hemoglobin below the lower reference value 5 and 8 dpi. (Figure S2a), the corresponding hematocrit value remained within the normal reference range (Figure S2b). The platelet counts of all four macaques were within the normal range throughout (Figure 5b), while total leukocyte counts were in the lower end of the reference range with subject 13428 dipping below normal 18 dpi (Figure 5c). In macaques 13422 and 150844, the majority of leukocytes were comprised of neutrophils at every tested time point (Figure 5d) although all white blood cell (WBC) counts remained within the normal range. Conversely, WBC counts of subject 13428, while also within normal ranges, were largely composed of lymphocytes (Figure 5e). All four macaques had mean corpuscular volumes (MCVs) and RBC distribution widths (RDWs) below and above reference ranges prior to infection, respectively (Figure S2c,e). These values did not deviate much from this baseline, although macaque 13422 was found to exhibit a jump in RDW on 18 dpi (Figure S2e).

### 3.3. Cytokine and Chemokine Response to Infection

**Figure 6:**
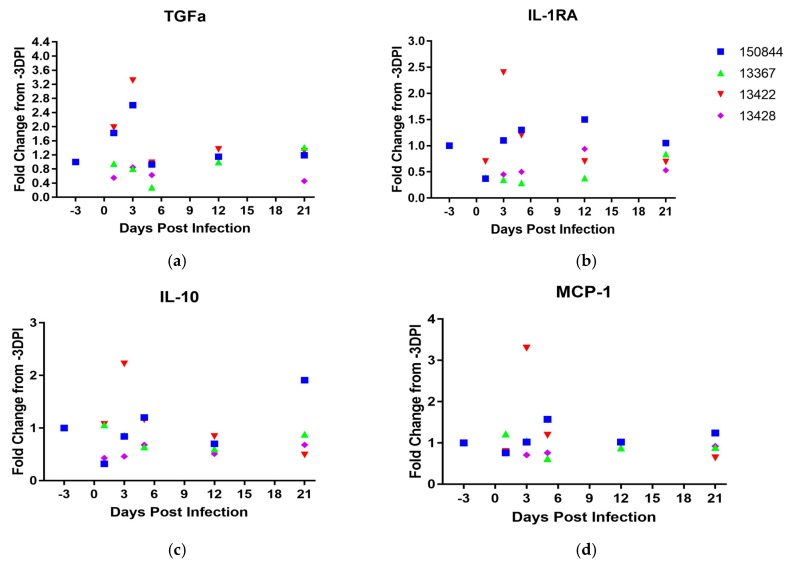
**Serum Cytokine Profile.** Sera taken from macaques on -3, 1, 3, 5, 12 and 21 dpi were subjected to multiplex analysis for cytokine levels. (**a**) TGFα, (**b**) IL-1RA, (**c**) IL-10, and (**d**) MCP-1. Macaque 150844 seen in blue, 13367 in green, 13422 in red and 13428 in purple. All data points represented as fold change relative to value observed 3 days prior to infection.

Systemic cytokine/chemokine levels were assayed in serum pre-exposure and at 1, 3, 5, 12, and 21 dpi. Infection with ZIKV did not produce substantial alterations when compared to baseline levels prior, with the exception of TGFα, IL-1RA, IL-10, and MCP-1 (Figure 6a–d). Macaque 13422 showed a striking increase in all four cytokines at 3 dpi. In all subsequent time points, macaque 13422 demonstrated a level of expression comparable to values observed prior to infection. Macaque 150844, however, also demonstrated similar patterns observed in 13422 albeit the spikes in IL-1RA, IL-10, and MCP-1 observed 3 dpi in 13422 were not observed (Figure 6b–d).

### 3.4. Histopathology

**Figure 7:**
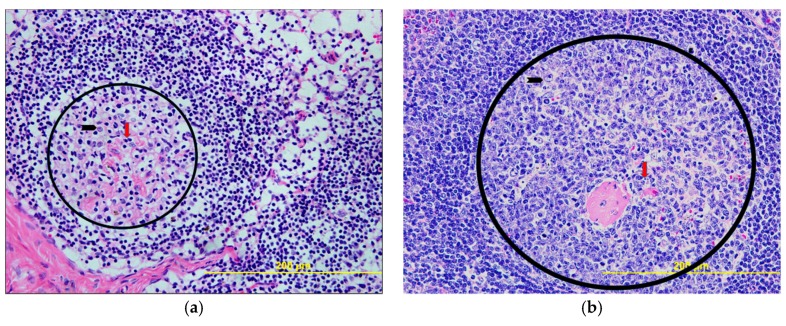

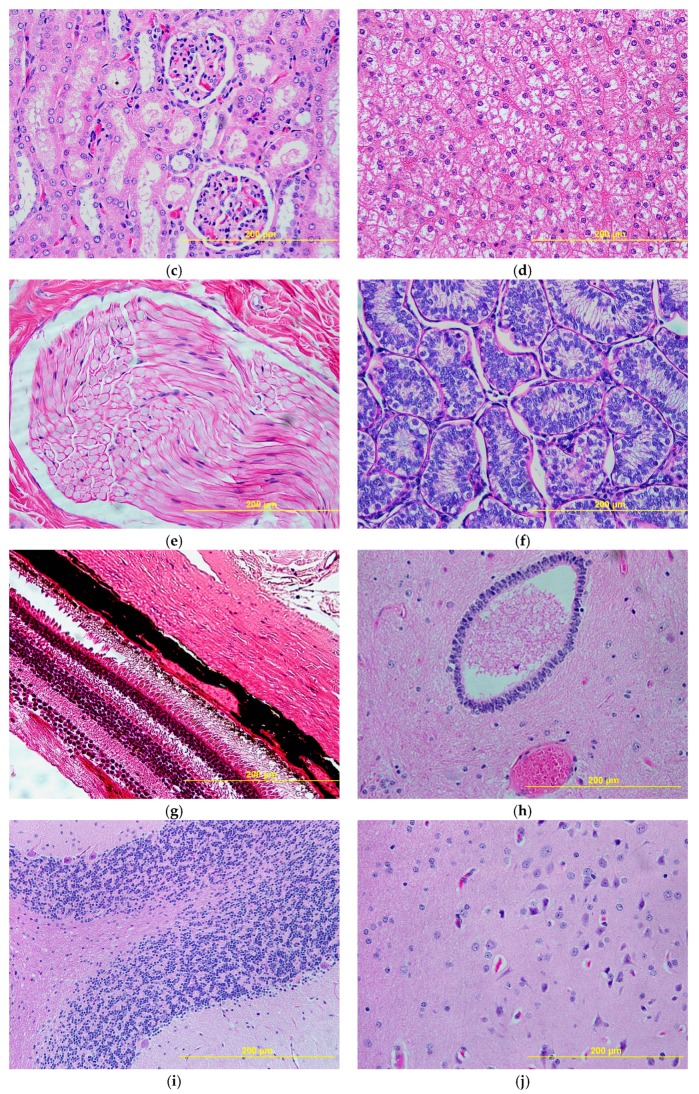
**Representative histology from infected cynomolgus macaques.** Upon necropsy, no histopathologic changes were noted, except for nonspecific changes related to systemic viremia in lymph nodes and spleen. (**a**) Lymph node: evidence of lymphoid activation and germinal center formation (black circle) with presence of activated lymphocytes, immunoblasts (black arrow) and apoptotic bodies in germinal centers (red arrow). (**b**) Spleen: evidence of lymphoid hyperplasia in periarteriolar lymphoid sheets with prominent germinal center formation (black circle) and presence of activated lymphocytes, immunoblasts (black arrow), and apoptotic bodies (red arrow) in the germinal centers. (**c**) Kidney: glomeruli and renal tubules do not reveal any pathologic changes. (**d**) Liver: hepatocytes are normally glycogenated. Cords and sinusoids do not reveal any evidence of inflammation. Portal triads (not shown) do not show any evidence of inflammation. (**e**) Nerve: spinal cord roots do not show any evidence of degeneration. Myelin stains (not shown) do not reveal any demyelination. (**f**) Testes: seminiferous tubules show normal spermatogenesis. No evidence of interstitial inflammation. (**g**) Eye: the retinal layers are normal. Pigment in the choroid layer is normal. (**h**) Spinal cord: no evidence of inflammation or neuronal cell death. Astrocytes and olygodendrocytes are normal. No evidence of microglial activation. (**i**) Cerebellum: molecular and granular layers are normal. Purkinje cells do not reveal any pathologic changes. (**j**) Cortical tissue of occipital lobe: no evidence of inflammation or neuronal cell death. Astrocytes and olygodendrocytes are normal. No evidence of microglial activation. All images taken at 40× magnification and 200 µm scale bar shown in each image.

Lymph nodes, spleen, kidneys, liver, eyes, testicles, cerebral cortex, cerebellar cortex, spinal cord and spinal nerve roots were examined upon necropsy (Figure 7). Lymph nodes and spleen (Figure 7a,b) showed non-specific changes related to the systemic viral infection that consisted of immune activation with presence of germinal centers in lymphoid follicles and periarteriolar lymphoid sheets in the spleen (Figure 7b). No other organs showed any histopathologic changes.

### 3.5. Mosquito Infectivity

To determine whether the viremia in these macaques reached sufficient levels to infect *Ae. aegypti*, cohorts of 50 mosquitoes were allowed to feed on the ears of anesthetized animals on 1–5, 10, 18, and 28 dpi. Engorged mosquitoes were incubated for 14 days then assayed for infection of the bodies (infection), legs (disseminated infection), and saliva (transmission potential). No infection was detected in any mosquito feeding on 1, 2, or 3 dpi. Subsequently, two of the four monkeys generated mosquito infections. Mosquitoes that fed on macaque 13422 on 4 dpi (viremia titer of 6.9 log_10_ genome copies/mL) developed 26% infection (11 positive bodies/42 engorged) (Table 1), with three of those infections disseminating to the legs, but with no virus detected in any saliva sample. The viremia in subject 13422 fell to ≈6.7 log_10_ genome copies/mL by day 5, and only 4.8% of mosquitoes that fed on this monkey on this day became infected, with no subsequent dissemination. Of mosquitoes that fed on subject 13367 at 4 dpi, with a viremia titer of 4.4 genome copies/mL, 2.4% became infected, with no dissemination.

To compare these findings to those of typical experimental infections, cohorts of *Ae. aegypti* were fed on artificial bloodmeals made with cynomolgus macaque erythrocytes and clone-derived ZIKV FSS13025 (Table 2). A contingency table revealed a significant effect of virus titer on the proportion of mosquitoes infected (DF = 4, χ^2^ = 51.0, *p* < 0.0001), with infection increasing as titer increased. Because of small expected values, we analyzed the effect of titer on the proportion of disseminated infections by grouping low (<1,000,000) and high (≥1,000,000) bloodmeal titers and comparing all disseminated infections arising from these titers; high titers did not result in a significantly higher proportion of disseminated infections (7.6%) than low titers (2.3%) (*p* = 0.45). The proportion of mosquitoes infected after feeding on the primates in vivo (26.2 % of mosquitos fed on monkey 13422 on day 4 pi at a titer of 6.9 log_10_ genome copies/mL) was similar to the proportion of mosquitoes infected via an artificial bloodmeal at a similar titer (20.8% of mosquitoes fed on 7.3 log_10_ FFU/mL) (*p* = 0.61).

## 4. Discussion

Results from this study largely support previous findings of minimal pathologic changes following ZIKV infection of cynomolgus macaques, with a lack of weight loss or febrile response (Figure 3a,b) [62,66]. Some rectal temperatures were low during the first 12 dpi (Figure 3b), as previously demonstrated in immunocompromised mice infected with ZIKV [82], as well as in bonnet monkeys (*Macaca radiatata*) infected with DENV4 [83]. Hypothermia results from our study may be partially explained by the effect sedation at the time of measurement. In general, both clinical chemistries and complete blood counts remained within normal limits. Interestingly, serum ALT levels rose in 2 primates (13367 and 13422) reaching levels close to the upper normal range, which may have been influenced by the high pre-infection levels (Figure 4d). Others have shown that serum ALT is elevated following ZIKV infection [63,64,66,71], in both rhesus and cynomolgus macaques as well as following DENV-2 infections in African green monkeys (*Chlorocebus aethiops*) [84]. Human disease does not necessarily present with elevated ALT levels but can in cases be associated with hepatic disease and coagulopathy [85].

In our study, viremia peaked between 2 to 5 dpi with maximum titers of 6.9 log_10_ genome copies/mL (13422) and 5.1 to 5.3 log_10_ genome copies/mL (13428, 13367 and 150844). Although direct comparisons of these results to other NHP studies is not possible due to differences in methodologies as well as the blood element assayed (i.e., whole blood vs serum vs plasma) [63,64,65,66,69,71], previous reports have demonstrated cynomolgus macaque viremia peaking between 5 log_10_ and 6.6 log_10_ genome copies/mL [62,66,71]. Human viral load has been found to range from 1 to 5 log_10_ genome copies/mL [50,51,52,53], with the larger values corresponding to serum samples from cutaneous capillary beds [51]. Of the four macaques we infected, 13422 demonstrated the highest viremia, which fell below the limit of detection (≈3.9 log_10_ genome copies/mL) by 10 dpi before a second peak of ≈4 log_10_ genome copies/mL was observed and sustained from 12–21 dpi (Figure 2). Bimodal viremia is a feature that has been described previously in both cynomolgus and rhesus macaques [62,63,64,69,75]. Given that the magnitude and duration of host viremia are critical elements to arboviral acquisition by vector mosquitos [86], standardizing subsequent studies to be more reflective of human disease will important. As most ZIKV viremia is reported in the context of symptomatic patients [50,51,52,53], in which viral burden is known to be significantly less than pre-symptomatic patients [87], it may be difficult for peak human viremia be identified. Nonetheless, as a significant determinant of mosquito infection [86], determining a more accurate human viremia profile will be critical in the development of subsequent animal and ecological models.

*Aedes aegypti* is accepted as the principal urban ZIKV vector [44,46,47]. Surprisingly, a very small proportion of *Ae. aegypti* that fed on the viremic macaques in this study became infected (Figure 2 and Table 1). Mosquitoes exposed to the highest viremia titer observed demonstrated a 26% infection rate, with none showing detectable virus in the saliva. To confirm that the population of *Ae. aegypti* used on the viremic macaques were susceptible, we provided them artificial bloodmeals made from of macaque blood. This experiment, like many others [86], showed that mosquito infection increased with increasing bloodmeal titer and that, with equivalent titers, monkeys and artificial bloodmeals infected similar proportions of mosquitoes (Table 2). These findings are consistent with a previous experiment undertaken by Dudley et al, in which a heavily colonized line of *Ae. aegypti* (black-eyed Liverpool, LVP) was fed on rhesus macaques at peak viremia (between 5.5 to 6.5 log_10_ genome copies/mL plasma) and only a single mosquito became infected but remained incapable of transmission following 25 days of extrinsic incubation [75]. Given the breadth and scale of the ZIKV outbreak in the Americas in 2015–2016, such poor competence of the major vector species is surprising. However, during a 1986 outbreak of yellow fever, *Ae. aegypti* was also shown to be relatively refractory despite its role as the principal vector [88]. This apparent conundrum likely reflects the superior behavioral and ecologic characteristics of this mosquito that contribute to its vectorial capacity despite limited vector competence.

A few inherent limitations of our study should deserve mention. First and foremost, the small number of macaques utilized necessitates follow-up analyses with a larger cohort to corroborate the findings, ideally performed in the context of both CHIKV-exposed and -naïve NHPs to discern whether the prior exposure played any role in the current findings. However, the >6-month delay between CHIKV exposure and ZIKV infection as well as the lack of evidence for cross-reactive immunity between CHIKV and ZIKV likely precluded the effects of CHIKV immunity on ZIKV pathogenesis. Second, only four (TGFα, IL-1RA, IL-10, and MCP-1) of 23 assayed serum cytokines demonstrated significant changes following ZIKV infection, similar to findings in related studies [69,89]. However, lacking uninfected controls, we could not discern whether these changes were due to infection or the immunomodulatory effects of repeated ketamine anesthesia [90]. Third, laboratory colonization is known to alter vector competence for arboviruses [91,92]. To minimize this confounding variable, we used F3 (viremic NHPs) and F4 (artificial bloodmeals) *Ae. aegypti*. Fourth, only a single strain of ZIKV was utilized, and it fairly well established that a given vector population can have widely different competency for disparate strains of the same virus, typified in the ZIKV vector competence literature [46,47,48]. Fifth, to preclude further cell culture passage of the FSS13025 ZIKV isolate and adaption for vector vs host [93,94], we utilized ZIKV FSS13025 infectious clone-derived virus. FSS13025 represents an Asian Lineage strain closely related to strains from the American outbreaks, and has been utilized experimentally by many groups; as a result, its phenotype in various systems has been well characterized [47,48,55,76,95,96,97,98]. Sixth, the present study used needle infection of the macaques, which may not adequately recapitulate natural infection involving mosquito transmission. A recent study in rhesus macaques exposed to ZIKV via mosquito transmission demonstrated a significantly altered pathogenesis when compared to needle infection, including altered viral kinetics, tissue tropism, and increased viral sequence heterogeneity [75]. These data suggest live mosquito transmission to macaques provides a compelling and highly relevant method to more closely model natural ZIKV infection. Seventh, the present study failed to recapitulate the detection of ZIKV in bodily fluid compartments, a common finding in human infections and in experimental infections of rhesus macaques [52,63,65,69,99]. To date, studies of ZIKV infection of cynomolgus macaques are far more limited than those performed in rhesus macaques, and of these, only two studies report detection of ZIKV in urine [62,66] in which urine was directly harvested from bladders via cystocentesis. During our study urine was harvested from waste pans daily, potentially decreasing yields of viable virus. Additionally, urine, tears, and saliva all were stored in carrier media upon harvesting, which may have led to virus being diluted below the limit of detection of qRT-PCR.

Human ZIKV infections can be affected by the cocirculation of other arboviruses (DENV, CHIKV) in endemic areas. Our macaques had all been exposed to CHIKV, a potential confounding element that could not be analyzed the absence of unexposed controls. However, such pre-exposure is nonetheless epidemiologically relevant due to the near simultaneous arrival of CHIKV and ZIKV in the Americas in 2013, with CHIKV causing major outbreaks in 2013-2014 before being overshadowed by ZIKV in 2015-2016 [100,101,102,103,104]. Given previously immunologically naïve populations involved, it not surprising that many concomitant and sequential infections have been reported [105,106,107].

In summary, our data indicate that in immunocompetent NHPs, ZIKV produces minimal pathologic changes and only transient viremia of titers that largely fail to efficiently infect *Ae. aegypti*. These findings underscore the need for further investigation of ZIKV and other arboviruses using NHPs and other hosts/reservoirs that develop viremias that reflect human infection. Given that in humans, asymptomatic patients and those with low DENV viremia are capable of experimentally infecting mosquitoes [108,109] a more robust analysis of *Ae. aegypti* and cynomolgus macaques is warranted. Finally, we utilized cynomolgus macaques (a potential sylvan reservoir species) and urban vectors *Ae. aegypti*, an epidemiologically imperfect combination that does not reflect ZIKV transmission; further studies with mosquito and mammalian hosts that more accurately model ZIKV circulation (e.g., feeding of the sylvatic *Ae. aegypti formosus* or *Ae. africanus* on viremic Old World primates, or *Sabethes* and *Haemogogus* spp fed on viremic New World primates) would be valuable going forward.

## Figures and Tables

**Table 1:** Mosquito infectivity of viremic cynomolgus macaques.

NHP ID	Day Post NHP Infection	Titer (log_10_ genomes/mL)	Number of Infected Mosquito Bodies/Total Fed	% Infected Mosquito Bodies/Total Fed
150844	1	3.4	0/34	0
150844	2	3.9	0/35	0
150844	3	4.8	0/34	0
150844	4	4.5	0/40	0
150844	5	5.2	0/33	0
13367	1	3.7	0/35	0
13367	2	4.9	0/25	0
13367	3	4.8	0/35	0
13367	4	4.4	1/41	2.4
13367	5	5.3	0/31	0
13422	1	4.7	0/35	0
13422	2	5.4	0/35	0
13422	3	5.5	0/36	0
13422	4	6.9	11/42	26.2
13422	5	6.7	2/42	4.8
13428	1	4.0	0/35	0
13428	2	5.0	0/27	0
13428	3	4.6	0/41	0
13428	4	4.7	0/28	0
13428	5	4.5	0/42	0

**Table 2:** Mosquito infectivity of cynomolgus macaque bloodmeal after 14 days extrinsic incubation.

Viral Titer (log_10_ genomes/mL)	Infection (# Infected Bodies/Total)	Total Dissemination (# Infected Legs/Total)	Dissemination from Infected Bodies (#Infected Legs/#Infected Bodies)
4.3	0% (0/28)	0% (0/28)	N/A
5.4	6.7% (1/15)	6.7% (1/15)	100% (1/1)
6.6	13.6% (3/22)	0% (0/22)	0% (0/3)
7.3	20.8% (10/48)	2.1% (1/48)	10% (1/10)
8.4	68.6% (24/35)	22.9% (8/35)	33.3% (8/24)

# is being used to denote “number”.

## Supplementary Materials

The following are available online at http://www.mdpi.com/1999-4915/10/11/661/s1, Figure S1: Sera was analyzed on Abaxis Comprehensive Diagnostic Profile rotors utilizing the VetScan VS2 system. Figure S2. Whole blood collected in K_2_EDTA tubes was utilized to perform CBC using the HemaVet 950 system.
