# Supplementary material for: ZIKV Demonstrates Minimal Pathologic Effects and Mosquito Infectivity in Viremic Cynomolgus Macaques
Source: Viruses. 2018 Nov 21;10(11):661. doi: 10.3390/v10110661 (PMC6267344; doi:10.3390/v10110661)
Supplement: Supplementary file 1 [file viruses-10-00661-s001.pdf]

viruses

MDPI

Supplementary Figure 1. Serum Biochemistries

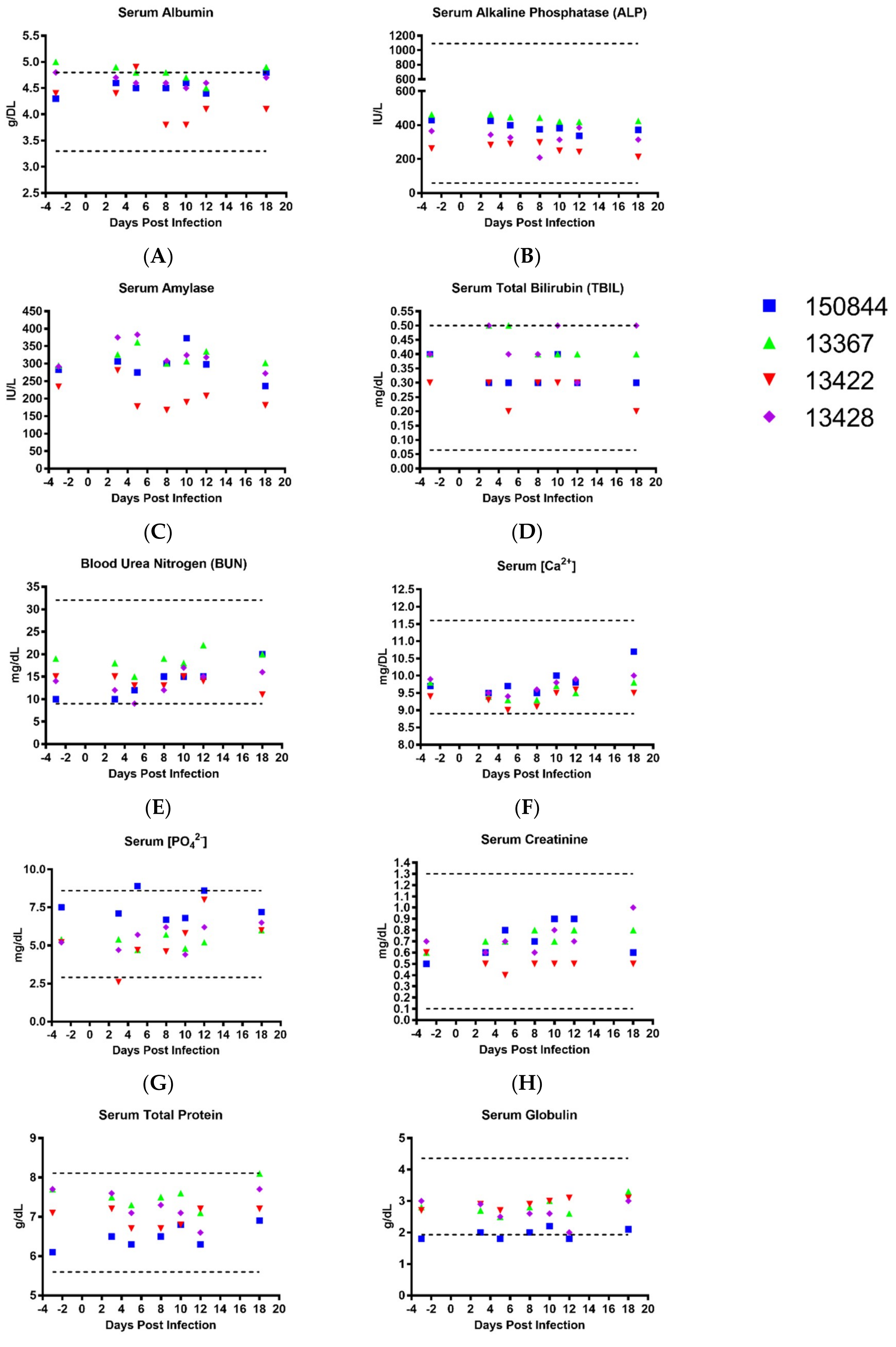

Figure S1. Sera was analyzed on Abaxis Comprehensive Diagnostic Profile rotors utilizing the VetScan VS2 system. **A**) serum albumin in g/dL, **B**) serum alkaline phosphatase in IU/L, **C**) serum amylase in IU/L, **D**) total serum bilirubin in md/dL, **E**) Blood urea nitrogen in mg/DL, **F**) serum Ca^{2+} in mg/dL, **G**) serum PO_4^{2-} in mg/dL, **H**) serum creatine in mg/dL, **I**) total serum protein in g/dL, and **J**) serum globulin in g/dL. Macaque 150844 seen in blue, 13367 in green, 13422 in red and 13428 in purple. Dotted lines represent upper and lower reference ranges for analytes where applicable.

Supplementary Figure 2. Additional HemaVet Readouts

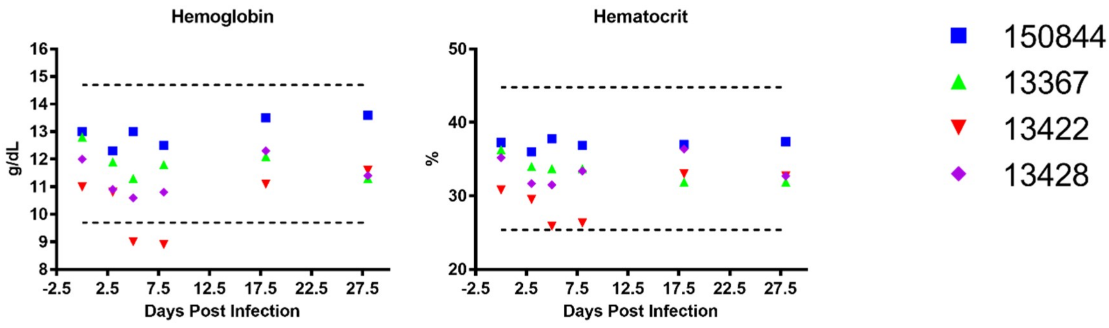

(**A**) (**B**)

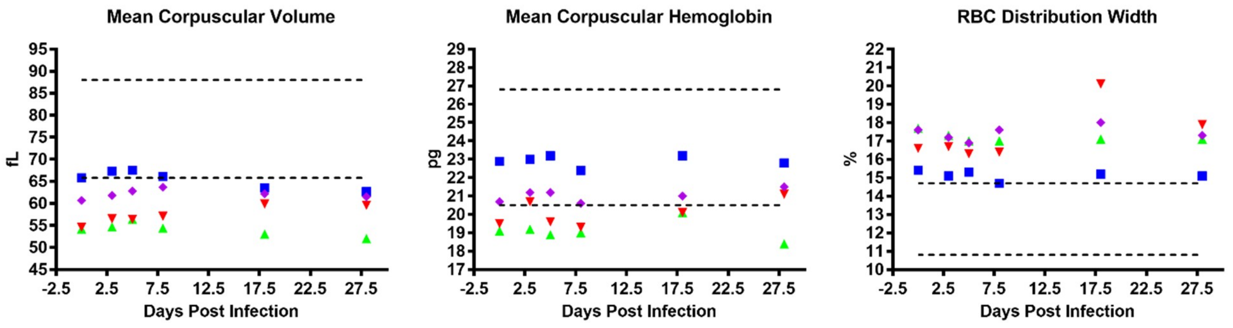

(**C**) (**D**) (**E**)

Figure S2. Whole blood collected in K_2EDTA tubes was utilized to perform CBC using the HemaVet 950 system. **A**) hemoglobin in g/dL, **B**) hematocrit (%), **C**) mean corpuscular volume (MCV) in femtoliters (fL), **D**) mean corpuscular hemoglobin in picograms, E) RBC distribution width (RDW) as a %. Macaque 150844 seen in blue, 13367 in green, 13422 in red and 13428 in purple. Dotted lines represent upper and lower reference ranges for analytes.
